# A comparative study of respiratory effects of erector spinae plane block versus paravertebral plane block for women undergoing modified radical mastectomy

**DOI:** 10.1186/s12871-024-02632-4

**Published:** 2024-07-30

**Authors:** Jehan Ahmed Sayed, Rasha Hamed, Abdelraouf MS Abdelraouf, Noha Yahia Mohammd El-hagagy, Mahmoud Bahaa El dean Mousa, Amani H. Abdel-Wahab

**Affiliations:** Anesthesia and Intensive Care Department, Faculty of Medicine, Assiut University, Asyut, Egypt

**Keywords:** Erector spinae plane block, Thoracic paravertebral block, Modified radical mastectomy, Pulmonary function

## Abstract

**Background:**

Inadequate acute postoperative pain control after modified radical mastectomy (MRM) can compromise pulmonary function. This work aimed to assess the postoperative pulmonary effects of a single-shot thoracic paravertebral block (TPVB) and erector spinae plane block (ESPB) in female patients undergoing MRM.

**Methods:**

This prospective, randomized comparative trial was conducted on 40 female American Society of Anesthesiologists (ASA) II-III, aged 18 to 50 years undergoing MRM under general anesthesia (GA). Patients were divided into two equal groups (20 in each group): Group I received ESPB and Group II received TPVB. Each group received a single shot with 20 ml volume of 0.5% bupivacaine.

**Results:**

Respiratory function tests showed a comparable decrease in forced vital capacity (FVC) and forced expiratory volume (FEV1) from the baseline in the two groups. Group I had a lower FEV1/FVC ratio than Group II after 6 h. Both groups were comparable regarding duration for the first postoperative analgesic request (P value = 0.088), comparable postoperative analgesic consumption (P value = 0.855), and stable hemodynamics with no reported side effects.

**Conclusion:**

Both ultrasound guided ESPB and TPVB appeared to be effective in preserving pulmonary function during the first 24 h after MRM. This is thought to be due to their pain-relieving effects, as evidenced by decreased postoperative analgesic consumption and prolonged time to postoperative analgesic request in both groups.

**ClinicalTrials.gov ID:**

NCT03614091 registration date on 13/7/2018.

## Introduction

Breast cancer affects almost 10% of women in their lifetime, making it the second most common type of cancer [1]. Surgery is the primary treatment for breast cancer, but over 30% of patients still experience inadequate pain control after surgery [2, 3].

Respiratory function can be affected after major surgical procedures, especially those performed on the thoracic and abdominal regions under general anesthesia. Postoperative pain, surgical trauma to the thoracic wall, and the application of a compressive chest dressing can limit chest movement and lung expansion, occasionally leading to hypoxemia [4].

During the 48 h following breast surgery, a decrease in the strength of the muscles used for breathing during inhalation and exhalation can be observed. This may be due to the incision made in the chest during the surgery, which can affect the ability of the muscles to generate pressure, thus altering the mechanics of the chest wall [5]. Additionally, the expiratory pressure may decrease immediately after surgery due to pain or the fear of experiencing pain [6]. 

The thoracic paravertebral block (TPVB) is a widely used technique for providing postoperative analgesia after breast surgeries [7]. Also, there is evidence suggesting that TPVB may have a positive impact on cancer recurrence after mastectomy [8], can prevent the transition from acute to chronic postoperative pain [9, 10], and reduces the occurrence of postsurgical neuropathic pain [11]. Erector spinae plane block (ESPB) is a technique described as an alternative to TPVB for providing thoracic analgesia. It reduced both acute and chronic pain occurrences similarly to TPVB following breast cancer surgery [12]. Several case reports and case series have described ESPB for managing acute and chronic thoracic pain [13]. 

We hypothesized that effective pain management using either ESPB or TPVB could help preserve respiratory function in women undergoing modified radical mastectomy (MRM).

The primary objective was to compare the effect of ESPB and TPVB on the forced vital capacity ((FVC) in females following MRM at the first postoperative 24 h. The secondary objectives were to compare the remaining respiratory function tests detected by the portable spirometer, dermatomal distribution, the visual numeric rating scale (VNRS), time to first rescue analgesia, total dose of analgesia requirement in the first postoperative 24 h, and the incidence of complications and side effects among the studied groups.

## Patients and methods

This prospective, single-center, randomized trial was conducted at Assiut University Hospital. The study protocol was approved by the local ethics committee of the Faculty of Medicine at Assiut University (IRB:17,200,238), registered on ClinicalTrials.gov (ID: NCT03614091) on 13/7/2018, and followed the Consolidated Standards of Reporting Trials (CONSORT) guidelines and the regulation of the Declaration of Helsinki. Forty female patients in the period between (February 2020 and April 2023), aged between 18 and 50, classified as American Society of Anesthesiologists (ASA) grade II-III, and undergoing modified radical mastectomy under general anesthesia, were included in the study after providing written informed consent. Patients with pre-existing infection at the site of the block, coagulopathy, body mass index (BMI) > 40 kg/m^2^, local anesthetic allergy, reduced pulmonary reserve, major cardiac disorders, renal dysfunction, pre-existing neurological deficits, or psychiatric illness were excluded from the study.

### Allocation

Forty female patients undergoing MRM were randomly divided into two groups using computer-generated random numbers, with 20 patients in each group to receive before general anesthesia either ESPB in group (I) or TPVB in group (II) using 20 ml of 0.5% bupivacaine in each block. The randomization process was carried out by a researcher who was not involved in the study, and the group allocation numbers were concealed in sealed opaque envelopes. These envelopes were opened only after the patients had been enrolled.

The attending anesthesiologist and anesthetic care unit (PACU) nurse were blinded to the block used.

During the preoperative visit, the study protocol, the procedure, and any possible side effects were explained to each patient, demographic data were recorded, and a verbal numeric rating scale (VNRS) was explained to patients. The patient was asked to rate their pain on a scale of zero to ten, with half integers allowed. Zero indicated no pain while ten represented the worst pain imaginable [14]. 

### Study protocol

All patients were kept fasting overnight and premedicated with alprazolam 0.25 mg and ranitidine 150 mg orally the night before and 2 h before surgery. Pulmonary function test (PFT) was performed for all of them on the day before operation. Forced vital capacity (FVC), forced expiratory volume in one second (FEV1), FEV1/FVC ratio, and peak expiratory flow rate (PEFR), were assessed via a portable spirometer (Enraf-Nonius, Model SPIRO 601 Medical Technologies) with the patient in the sitting or semi-recumbent position. Pulmonary function was repeated at 6,12, and 24 h postoperatively.

In the pre-operating room before the surgery, standard monitoring such as non-invasive blood pressure, ECG, and pulse oximetry were connected before performing the block. The blocks were performed under all aseptic precautions with infiltration of local anesthesia using a 22-gauge echogenic needle (Pajunk, sonoplex stim cannula, Geisingen, Germany; 80 mm) and the ultrasound machine (Madison X6) with high-frequency linear array probe (38 mm,7–12 MHz frequency). The blocks were performed by the same anesthetist not involved in the preoperative or postoperative assessment of the patients, anesthesia management, or data collection.

During the ESPB at the level of T4–T5, the patient was seated, and an ultrasound scan was performed to locate and mark the targeted thoracic spine level. The identification process involved counting the ribs from above. The skin was then sterilized using 2% chlorhexidine in a 70% alcohol solution. The ultrasound transducer was placed transversely to locate the spinous process, lamina, and transverse process. The tip of the transverse process was centered on the ultrasound screen, and the transducer was then rotated 90 degrees into a longitudinal orientation to obtain a parasagittal view. The ultrasound image identified 2 or 3 hypoechoic muscle layers on the transverse processes’ tip depending on the level. From T1 to T5, the erector spinae, rhomboid major, and trapezius muscles are visible, positioned posteriorly, and superficially to the transverse processes. The rhomboid major muscle’s lower border was present at the level of T5 or T6, and the erector spinae and trapezius muscles were visible at more caudal levels. To place the needle tip between the posterior fascia of the erector spinae and the targeted transverse process’s tip, an 8 cm 22-gauge block needle was inserted in-plane to the ultrasound beam in a cephalad-to-caudad direction. After confirming the tip’s position by injecting 0.5 mL of 0.5% bupivacaine and visualizing linear fluid spread deep to the erector spinae muscle, a total of 20 mL of 0.5% bupivacaine was injected [15]. 

TPVB was administered at the T4 level while the patient was in a sitting position. The skin was sterilized with 2% chlorhexidine in a 70% alcohol solution. The ultrasound probe was placed along the midline in a sagittal position. The spinous processes were identified, and then the probe was moved parallel to the vertebral column until the acoustic window between the transverse processes was located. Next, the transverse process, erector spinae muscles, costotransverse ligament, sliding hyperechoic pleura, and adjacent paravertebral space were identified. Finally, an echogenic 22-gauge block needle was inserted and 20 ml of bupivacaine 0.5% was deposited between the pleura and the costotransverse ligament [16]. 

After performing the blocks, we assessed their success every five minutes for 30 min by evaluating the loss of pinprick sensation for the sensory block and the inability to perceive the cold sensation of ethanol alcohol for the autonomic block within T1 to T8 dermatomal distribution. We recorded the total number of blocked dermatomes, and if the patient did not lose sensation in at least one segment, the block was considered unsuccessful and was excluded from the study. Additionally, we observed the incidence of complications such as pneumothorax and surgical emphysema.

In the operating room general anesthesia was induced with intravenous (IV)fentanyl 1.5 µg/kg, propofol 1.5–2 mg/kg, and tracheal tube was facilitated with 0.15 mg/kg cis-atracurium. Anesthesia was maintained with 1-1.5 MAC isoflurane in 50% air: oxygen mixture. Volume-controlled mechanical ventilation was adjusted to maintain end-tidal CO_2_ between 30 and 35 mmHg. Muscle relaxation was maintained during surgery using 0.03 mg/kg bolus doses of Cis-atracurium, using the train of four monitoring. The heart rate (HR) and mean blood pressure (MBP) were recorded before induction, after induction, after tracheal intubation, at skin incision, and then every 30 min until the end of surgery. All patients received intraoperative IV 4 mg of ondansetron and one gram of paracetamol. At the end of the surgical procedure, all anesthetic agents were ceased. The inspired oxygen fraction (FiO_2_) was increased to 1.0 and once two twitches on the train of four were detected, the muscle relaxant was reversed. The patients were extubated and transferred to the PACU after regaining consciousness from the anesthesia. At the PACU, the hemodynamic state was recorded (MBP and HR), assessment of analgesia at (0.5, 1,2, 4, 6, 8, 12, 24 h postoperative), and pulmonary function at 6, 12, and 24 h postoperatively. Observations were performed by a PACU nurse blinded to the block used. IV 0.1 mg/kg nalbuphine was used as rescue analgesia whenever the pain score (VNRS) ≥ 4.

### Sample size calculations

Based on a previous study [17], power calculation estimated that to detect an effect size of 1.27 difference between means of FVC of two independent groups, with a p-value < 0.05 and 95% power, a sample size of 36 patients was needed (G Power 3.1). To overcome patients’ dropouts, we enrolled 40 patients.

## Statistical analysis

Data entry and analysis were done using SPSS version 22 (Statistical Package for Social Science). Data were presented as frequency, mean, standard deviation, median, and range. Chi-square and Fisher’s Exact tests were used to compare qualitative variables. Independent samples t-test was used to compare quantitative variables between groups. Repeated measures ANOVA with Bonferroni correction for within-group comparison in each group in case of parametric data. Mann-Whitney test was used to compare quantitative variables between groups. Friedman test, 2-way ANOVA/ Multiple comparisons were done for within-group comparison in each group in case of non-parametric data. The P-value is considered statistically significant when *P* < 0.05.

## Results

**Fig. 1:**
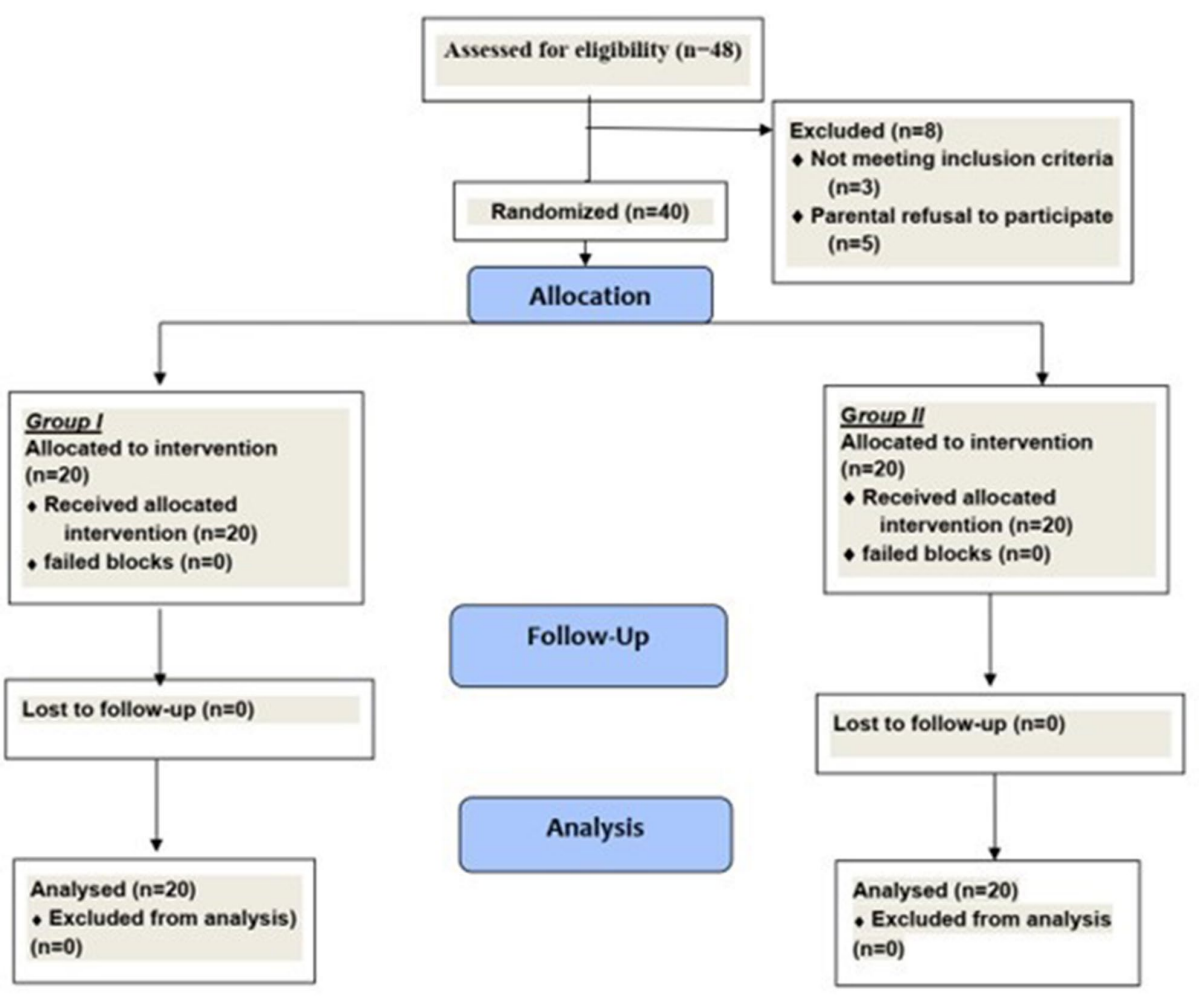
Participant flow diagram

A total of forty-eight patients were enrolled in the study. Eight participants were excluded, leaving twenty patients analyzed in each group (Fig. 1).

The demographic and clinical characteristics of the patients were similar between the studied groups. Dermatomal distribution was similar in both groups, except at the T1 level (P value = 0.008) (Table 1).

**Table 1:** Patients’ demographics and clinical data of the studied groups

Personal data	Group I (n = 20)	Group II (n = 20)	P-value
Age (years)	49.95 ± 13.15	45.35 ± 11.03	0.238
ASA: II/ III	13/7	15 /5	0.490
Weight (kg)	75.15 ± 12.24	75.85 ± 10.16	0.845
Height (cm)	161.70 ± 6.33	166.15 ± 8.44	0.067
BMI (kg/m²)	28.86 ± 5.24	27.69 ± 4.63	0.461
Hospital length of stay (days)	3.80 ± 1.06	3.70 ± 1.26	0.787
Anesthesia time (min)	119.15 ± 7.80	117.95 ± 7.39	0.620
Operative time (min)	73.30 ± 10.15	77.65 ± 10.20	0.184
Recovery time (min)	14.20 ± 2.67	15.30 ± 2.92	0.221
No. of dermatomes	5.35 ± 1.14	4.90 ± 0.85	0.855
Dermatomal distribution			
T1	7	0	0.008*
T2	11	13	0.519
T3	20	20	--
T4	20	20	--
T5	20	20	--
T6	19	19	1.000
T7	9	6	0.327
T8	1	0	1.000
Time to first rescue analgesia:(hours)	10.70 ± 3.50	12.85 ± 4.23	0.088
Total analgesic consumption(mg)	8.70 ± 2.18	8.00 ± 2.34	0.855

Data are presented as mean ± SD or as numbers. *Abbreviations* SD, standard deviation, American Society of Anesthesiologists (ASA), body mass index (BMI), number (NO). *P* < 0.05 indicates statistically significant differences

Respiratory function tests: regarding FVC and FEV1 there were statistically significant mild decreases from the baseline at each time interval (6, 12, and 24 h) and did not return to baseline levels. this decrease in FVC and FEV1 had no statistically significant difference between the studied groups. The FEV1/ FVC ratio was statistically significant lower in group I than in group II only after 6 h (P value = 0.0.044). Also, PEFR was statistically significantly lower in group II than in group I after 6 h, 12 h, and 24 h (P value 0.002, 0.008, and 0.000 respectively) (Table 2).

**Table 2:** Postoperative pulmonary function of the studied groups

Post-operative spirometry	Group I (n = 20)	Group II (n = 20)	P-value^1^	P-value^2^	P-value^3^
Baseline	3.41 ± 0.35	3.55 ± 0.47	0.281	-	**-**
6 h	3.08 ± 0.42	2.96 ± 0.46	0.412	0.002*	0.000*
12 h	3.10 ± 0.48	3.12 ± 0.44	0.874	0.004*	0.000*
24 h	3.21 ± 0.41	3.15 ± 0.46	0.666	0.000*	0.000*
FEV1(L):					
Baseline	3.21 ± 0.35	3.25 ± 0.37	0.738	-	-
6 h	2.85 ± 0.52	3.00 ± 0.45	0.326	0.000*	0.002*
12 h	2.97 ± 0.48	2.82 ± 0.33	0.283	0.005*	0.000*
24 h	2.98 ± 0.47	2.87 ± 0.35	0.397	0.000*	0.000*
FEV1/ FVC ratio:					
Baseline	0.94 ± 0.07	0.92 ± 0.09	0.403	-	-
6 h	0.93 ± 0.13	1.02 ± 0.16	0.044*	0.646	0.005*
12 h	0.97 ± 0.15	0.91 ± 0.10	0.196	0.497	0.780
24 h	0.93 ± 0.09	0.92 ± 0.11	0.768	0.528	0.997
PEFR(L/s):					
Baseline	5.20 ± 1.09	4.74 ± 1.02	0.180	-	-
6 h	5.08 ± 1.12	4.16 ± 0.57	0.002*	0.543	0.006*
12 h	5.17 ± 1.12	4.29 ± 0.85	0.008*	0.907	0.063
24 h	5.26 ± 1.23	3.83 ± 0.69	0.000*	0.770	0.000*

Data are presented as mean ± SD *Abbreviations* standard deviation (SD), forced vital capacity (FVC), Forced expiratory volume (FEV1), and Peak expiratory flow rate (PEFR). *P* < 0.05 indicates statistically significant differences. P-value^1^: Comparison between Groups. P-value^2^: Comparison with Baseline in Group I, P-value3: Comparison with Baseline in Group II

The time of the first analgesic request (P value = 0.088) and total postoperative analgesic dose (P value = 0.855) were comparable in the two groups (Table 1).

Postoperative VNRS scores were statistically significant differences between the studied groups at various time intervals after 0.5 h, 1 h, 2 h, 4 h, and 8 h (P value: 0.018*, 0.000*, 0.001*, 0.003*, 0.019* respectively). The VNRS score did not exceed 4 in either of the groups during the studied time intervals except at 24 h where the range of VNRS reached 6 in the two groups (P value: 0.092) (Table 3).

**Table 3:** postoperative VNRS

VNRS	Group I (n = 20)	Group II (n = 20)	P-value^1^	P-value^2^	P-value^3^
0.5 h.	0.0 (0.0–1.0)	0.0 (0.0–0.0)	0.018*	-	-
1 h.	0.5 (0.0–1.0)	0.0 (0.0–0.0)	0.000*	0.583	1.000
2 h.	1.0 (0.0–1.0)	0.0 (0.0–1.0)	0.001*	0.165	0.519
4 h.	1.0 (1.0–2.0)	1.0 (0.0–2.0)	0.003*	0.002*	0.057
6 h.	2.0 (1.0–2.0)	2.0 (1.0–2.0)	0.333	0.000*	0.000*
8 h.	2.0 (2.0–4.0)	2.0 (1.0–4.0)	0.019*	0.000*	0.000*
12 h.	4.0 (2.0–4.0)	3.0 (1.0–4.0)	0.388	0.000*	0.000*
24 h.	4.0 (4.0–6.0)	4.0 (2.0–6.0)	0.092	0.000*	0.000*

Data are presented as Median and Range. *Abbreviations* visual numeric rating scale (VNRS), hour (hr.). *P* < 0.05 indicates statistically significant differences. P-value^1^: Comparison between Groups. P-value^2^: Comparison with 0.5 h.in Group I, P-value3: Comparison with 0.5 h. in Group II

**Fig. 2:**
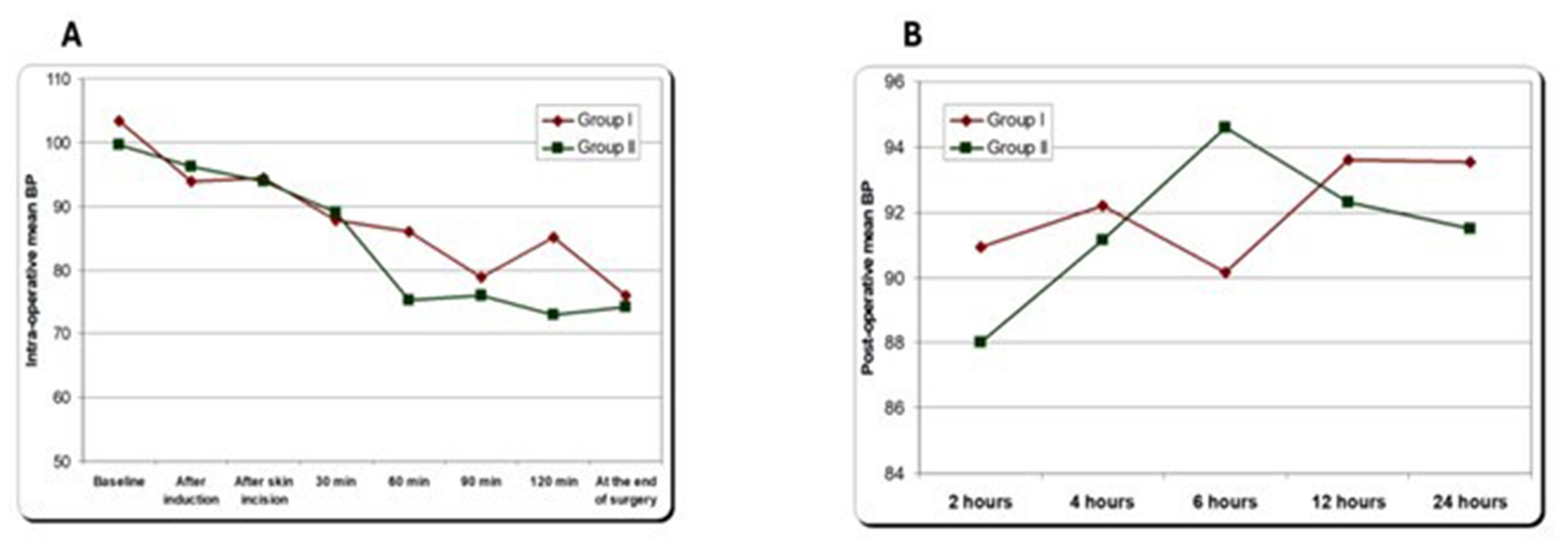
**A**: intraoperative MAP among studied groups, **B**: postoperative MAP among studied groups. mean blood pressure (MBP). Data expressed as mean ± SD. *P* < 0.05 significant difference

**Fig. 3:**
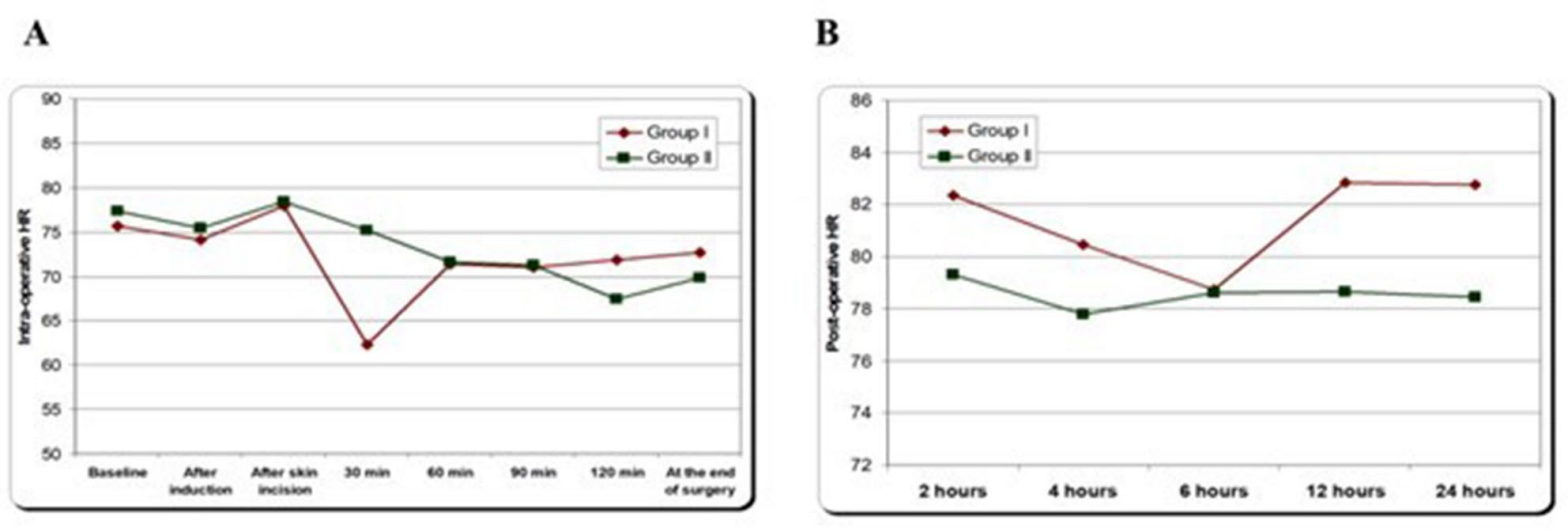
**A**: intraoperative heart rate among studied groups, **B**: postoperative heart rate among studied groups. Data expressed as mean ± SD. *P* < 0.05 significant difference

All patients in both groups maintained stable hemodynamics and no patients required vasopressor intraoperative or in the first postoperative 24 h (Fig. 2 and 3).

There were no complications or side effects observed in either group of patients.

## Discussion

In this study, we evaluated the respiratory and analgesic benefits of TPVB and ESPB in patients who underwent modified radical mastectomy. Our findings showed that both TPVB and ESPB are effective in managing postoperative pain, with similar respiratory effects, comparable duration for the first request of postoperative analgesia, comparable post-operative analgesic consumption, and stable hemodynamics with no reported side effects.

Vargas M et al. reported an intraoperative reduction in the functional residual capacity (FRC) of the lungs in the absence of pulmonary comorbidities occurs at the induction of anesthesia and remains stable intraoperatively [18]. Several mechanisms can exacerbate FRC reduction during anesthesia and after surgery, such as the decreased diameter of the chest wall, changes in the diaphragmatic shape and position, and redistribution of the thoracic blood volume. A reduction in the thoracic diameter is related to a reduction in the inspiratory muscular tone, which could cause alterations in chest wall recoil properties. Furthermore, compressive dressings used at the end of breast surgery contribute to decreasing the chest wall movement by decreasing the internal diameter of the rib cage, which decreases the lung volume. Reportedly, the loss of muscle tone and an increase in the intraabdominal pressure could favor a cephalic shift of the diaphragm, contributing to a further reduction in the functional residual capacity [4, 18, 19]. All of the above reasons may have contributed to or caused mild differences between postoperative and preoperative spirometry values in our patients. These changes reflected a restrictive ventilatory pattern, including a decrease in FVC and reduction in FEV1 with a nearly normal FEV1/FVC ratio which reflects a reduction in the chest wall distensibility and decreased expiratory effort [20]. Our study found that improved pain control, as indicated by a decrease in the total requirement for postoperative analgesia over the first 24 h, may have contributed to the preserved respiratory function in all patients studied. There was no significant difference between the two groups in terms of FVC, FEV1, or FEV1/FVC ratio. This confirms that ESPB is effective as the paravertebral plane block for the preservation of pulmonary function but there is a significant difference between both groups regarding PEFR showing improvement of pulmonary function in the ESPB group than PVB group, PEFR may have been inhibited by unilateral partial intercostal nerve block as weakness in the intercostal muscles is known to be induced by epidural local anesthetic (although overall lung volumes are minimally affected) [21]. 

In agreement with this study, Matyal et al. conducted a study on the effect of TPVB on pulmonary function in patients undergoing video-assisted thoracoscopic surgery, and they found that TPVB was associated with better preservation of pulmonary function [22].

In agreement with our study, Yildiz M et al. found preservation in FEV1 and FVC in the ESPB group in comparison to the control group at 2 and 24 h after surgery (*p* < 0.05 in each). FEV1/FVC and PEFR values were similar in each time interval in patients undergoing Laparoscopic cholecystectomy [23].

The present study showed no significant difference in nalbuphine consumption in both groups. However, the two groups were comparable regarding VNRS, time to first analgesic request. There were no complications in either of the blocks, and they were both safe. In agreement with our results Xiong C et al., metanalysis reported that the postoperative analgesic effects of PVB versus ESPB are distinguished by the surgical site. For breast surgery, the postoperative analgesic effects of PVB and ESPB are similar. For thoracic surgery, the postoperative analgesic effect of PVB is better than that of ESPB [20].

The number of segments blocked in TPVB influenced by the location of the needle tip during injection which influences the spread of LA [24], TPVB requires more careful needle handling and advancement of a needle for a longer distance to the target [25]. Also, the presence of the endothoracic fascia within the thoracic paravertebral space (TPVS) has been found to affect the spreading pattern of nerve blocks. Injecting in the more dorsal part of TPVS results in a more localized cloud-like spread, while injecting in the ventral part of TPVS results in a more desirable longitudinal spreading pattern. Identifying the endothoracic fascia by ultrasound is difficult, and its presence may have influenced the spread of the LA drug and the number of blocked segments. The spread of injectate in the craniocaudal direction is limited with TPVB, multiple level injections are recommended and conducted in many institutions for breast surgery which sometimes affects an extensive dermatomal area (from T1 to T7) [24]. 

Both groups in the current study exhibited similar levels of dermatomal block between T2 and T5. In Group I, the block extended to the T7 dermatome in 9 (45%) patients, compared to 6 (30%) in group II. In group I, T1 dermatome was blocked in 7 patients, T8 was blocked in one patient, while no patients in group II exhibited these blockages. The difference between the two groups was found to be statistically insignificant. These results were similar to Singh et al. who found that both ESPB and PVB had similar levels of dermatomal block between T2 and T6. However, in the ESPB group, the block extended to the T7 dermatome in 9 (30%) patients, compared to none in the TPVB group. The difference between the two groups was statistically insignificant (*P* = 0.5) [26]. 

Both groups in our study had intraoperative and postoperative hemodynamic stability. This is in agreement with previous studies that demonstrated that patients with either PVB or ESPB had a stable hemodynamic profile despite the sympathetic block [27, 28]. Also, in a study by Helal et al., it was found that PVB showed more hemodynamic stability in terms compared to thoracic epidural in perioperative management for mastectomy, with comparable pain control [29].

Although ESPB has an advantage over TPVB in terms of ease of performance and safety due to the absence of vascular structures and pleura in the immediate vicinity [30], no side effects were reported in either group in our study. This may be attributed to the use of ultrasound, which makes the performance of TPVB easier while also decreasing the incidence of complications such as vascular puncture, nerve injury, and pneumothorax that are associated with blind techniques [31, 32]. 

### Limitations

The current study has several limitations. It was a single-center study with a small sample size and lacked a control group that did not receive any block, making it difficult to compare the degree of effectiveness of each block in preserving pulmonary function. Additionally, the study did not employ blinding and did not assess VNRS at movement. Also, the study duration was limited to the first 24 h after surgery, so it did not evaluate the pulmonary function and analgesic consumption beyond this time frame (48 h &72 h) or the effect of each block on the incidence of chronic pain. So, we recommend further larger multicenter studies with a longer duration of follow-up.

## Conclusion

Based on our study, we found that both ultrasound guided ESPB and TPVB appeared to be effective in preserving pulmonary function during the first 24 h after MRM. This is thought to be due to their pain-relieving effects, as evidenced by decreased postoperative analgesic consumption and prolonged time to first rescue analgesia in both groups.

## Data Availability

The datasets are available from the corresponding author upon reasonable request.

## Abbreviations

MRM: Modified radical mastectomy
TPVB: Thoracic paravertebral block
ESPB: Thoracic Erector spinae plane block
(ASA: American Society of Anesthesiologists
GA: General anesthesia
FVC: Forced vital capacity
FEV1: Forced expiratory volume in one second
PEFR: Peak expiratory flow rate
VNRS: Visual numeric rating scale
BMI: Body mass index
IV: Intravenous
HR: Heart rate
MBP: Mean blood pressure
FiO_2_: Inspired oxygen fraction
PACU: Post-anesthetic care unit
FRC: Functional residual capacity
